# Cost-effectiveness of fibronectin testing in a triage in women with threatened preterm labor: alleviation of pregnancy outcome by suspending tocolysis in early labor (APOSTEL-I trial)

**DOI:** 10.1186/1471-2393-9-38

**Published:** 2009-09-01

**Authors:** Jolande Y Vis, Femke F Wilms, Martijn A Oudijk, Martina M Porath, Hubertina CJ Scheepers, Kitty WM Bloemenkamp, Annemiek C Bolte, Jérôme Cornette, Jan B Derks, Johannes J Duvekot, Jim van Eyck, Anneke Kwee, Brent C Opmeer, Maria G van Pampus, Fred K Lotgering, Sicco A Scherjon, Krystyna M Sollie, Marc EA Spaanderman, Christine Willekes, Joris AM van der Post, Ben Willem J Mol

**Affiliations:** 1 Department of Obstetrics and Gynaecology, Academic Medical Centre, Amsterdam, The Netherlands; 2 Department of Obstetrics and Gynaecology, Máxima Medical Centre, Veldhoven, The Netherlands; 3 Department of Obstetrics and Gynaecology, Maastricht University Medical Centre, Maastricht, The Netherlands; 4 Department of Obstetrics and Gynaecology, Leiden University Medical Centre, Leiden, The Netherlands; 5 Department of Obstetrics and Gynaecology, VU Medical Centre, Amsterdam, The Netherlands; 6 Department of Obstetrics and Gynaecology, Erasmus Medical Centre, Rotterdam, The Netherlands; 7 Department of Obstetrics and Gynaecology, University Medical Centre, Utrecht, The Netherlands; 8 Department of Obstetrics and Gynaecology, Isala Clinics, Zwolle, The Netherlands; 9 Department of Clinical Epidemiology, Biostatistics and Bioinformatics, Academic Medical Centre, Amsterdam, The Netherlands; 10 Department of Obstetrics and Gynaecology, University Medical Centre, Groningen, The Netherlands; 11 Department of Obstetrics and Gynaecology, Radboud University Nijmegen Medical Centre, Nijmegen, The Netherlands

## Abstract

**Background:**

At present, women with threatened preterm labor before 32 weeks of gestation are, after transfer to a perinatal center, treated with tocolytics and corticosteroids. Many of these women are treated unnecessarily. Fibronectin is an accurate predictor for the occurrence of preterm birth among women with threatened preterm labor. We will assess whether triage of these women with fibronectin testing, cervical length or their combination is cost-effective.

**Methods/Design:**

We will investigate a prospective cohort of women referred to a perinatal centre for spontaneous threatened preterm labor between 24 and 34 weeks with intact membranes. All women will be tested for fibronectin and cervical length. Women with a cervical length <10 mm and women with a cervical length between 10-30 mm in combination with a positive fibronectin test will be treated with tocolytics according to local protocol. Women with a cervical length between 10-30 mm in combination with a negative fibronectin test will be randomised between treatment with nifedipine (intervention) and placebo (control) for 48 hours. Women with a cervical length > 30 mm will be managed according to local protocol. Corticosteroids may be given to all women at the discretion of the attending physician. Primary outcome measure will be delivery within 7 days. Secondary outcome measures will be neonatal morbidity and mortality, complications of tocolytics, costs and health related quality of life. The analysis will be according to the intention to treat principle. We anticipate the probability on preterm birth within 7 days in the group of women with a negative fibronectine test to be 5%. Two groups of 110 women will be needed to assure that in case of non-inferiority the difference in the proportion of preterm deliveries < 7 days will be within a prespecified boundary of 7.5% (one sided test, β 0.2, α 0.05). Data obtained from women with a positive and negative fibronectin tests in both the cohort study and the trial will be integrated in a cost-effectiveness analysis that will assess economic consequences of the use of fibronectin.

**Discussion:**

This study will provide evidence for the use of fibronectin testing as safe and cost-effective method in a triage for threatened preterm labor.

**Trial registration:**

Nederlands Trial Register (NTR) number 1857, .

## Background

Preterm birth is the most frequent cause of perinatal mortality and severe perinatal morbidity in the Western world [1]. Preterm birth can be preceded by rupture of the membranes and/or by uterine contractions leading to cervical effacement and dilation. Current treatment of women with threatened preterm labor is tocolysis to diminish contractions and the administration of corticosteroids to enhance fetal lung development [2]. In the Netherlands, 7.9% of all deliveries are preterm, and 1.6% of all neonates are born before 32 weeks of gestation [3]. Women at risk for preterm birth prior to 32 weeks are transferred to a perinatal centre [4].

Discriminating those women who will deliver preterm is difficult: about half of the women treated with tocolytics and/or corticosteroids do not deliver within a short time after the occurrence of symptoms. As tocolytics and corticosteroids can cause severe side-effects in both mother and child [5-8], concerns have been expressed about overtreatment. If we are able to identify women that eventually do not deliver preterm and thus do not benefit from referral and treatment, we could prevent them from being exposed to the potential side effects of tocolytics and corticosteroids. In addition, management costs could be reduced by preventing unnecessary transport of pregnant women to perinatal centers and the associated higher treatment costs.

Fetal fibronectin is an internationally accepted predictor of preterm birth [9]. Fetal fibronectin is a glycoprotein found in amniotic fluid, placental tissue, and the extracellular substance of the decidua next to the placental intervillous space. It is thought to be released through mechanical or inflammatory mediated damage to the membranes or placenta before birth [10]. Swabs can be taken from the ectocervix or posterior vaginal fornix, and an enzyme linked immunosorbent assay (ELISA) containing FDC6 monoclonal antibody can be used to detect fetal fibronectin [11]. The results may indicate the likelihood of spontaneous preterm birth [12]. In clinical use, however, factors such as contamination of the sample with maternal blood, sampling within 24 hours after intercourse, and preeclampsia may reduce the accuracy of the test and give false positive results [13-15].

The predictive capacity of fibronectin has been the topic of previous studies. Honest and co-workers published in 2002 a systematic review on the predictive capacity of fibronectin for preterm delivery [16]. They identified 40 studies reporting on symptomatic women. The summary likelihood ratio for a positive result was 5.4 (95% CI 4.4 to 6.7) for predicting birth within 7 to 10 days of testing, with a corresponding likelihood ratio for a negative result of 0.25 (95% CI 0.20 to 0.31). Therefore fibronectin testing might be able to reduce unnecessary treatment and subsequent costs. On the other hand, introducing fibronectin tests has not yet proven to reduce health care costs if the subsequent management is subject to physicians preferences [17-21]. In addition, high false positive rates may increase health care costs in the management of low risk women.

For cervical length measurement, the situation is different. This test is shown to predict preterm delivery in asymptomatic women at 20 weeks [22]. However, the accuracy of the test is only marginally evaluated in women with symptoms of preterm labor at a later gestational age [23]. Gomez et al. evaluated the predictive accuracy of a combination of fetal fibronectin measurement and cervical length measurement [24]. In their study, 15% of the women had cervical lengths below 15 mm, and one third of these women delivered within 48 hours. Among those with a cervical length between 15 mm and 30 mm (36% of the population), 25% had a positive fibronectin test, and 15% of this group delivered within 48 hours. Of the remaining 75% of the women, less than 5% delivered within 7 days. These results are comparable with our pilot study of Wilms et al. Amongst 19 women with a cervical length between 10-30 mm and a negative fibronectin test, only 1 woman delivered within 7 days[25].

At present, fibronectin testing is not routinely used in The Netherlands. To assess the risk for threatened preterm labor, only cervical length is routinely measured[2]. This measurement is frequently used as an argument to transfer and treat women for threatened preterm labor, even if clinical symptoms are mild. Identifying women that are at high risk for preterm delivery could benefit from adding fibronectin testing. Therefore we will evaluate if adding fibronectin testing to cervical length measurement is a safe and cost-effective strategy as a triage for women with threatened preterm labor.

## Methods/Design

### Aims

We will evaluate a risk assessment with fibronectin testing for women with clinical signs of threatened preterm labor. We will assess whether triage of these women with fibronectin testing, cervical length measurements or a combination is cost-effective. The results of this study can be used to support a policy of expectant management without immediate referral to a neonatal centre and without tocolysis in women with a low risk for preterm birth.

### Participants/eligibility criteria

All women between 24 and 34 weeks of gestational age with primary complaints associated with preterm labor and intact membranes will be eligible for inclusion in the study. Women with vaginal bleeding, a cerclage, cervical dilatation of more than 3 cm or previous treatment for threatened preterm labor in the current pregnancy are excluded. Other exclusion criteria are hypertension, contra indications for nifedipine and fetal distress or maternal conditions that may lead to pregnancy termination within 7 days. The study will be limited to women admitted to a perinatal centre with neonatal intensive care facilities.

### Procedures, recruitment, randomisation and collection of baseline data

Women meeting the eligibility criteria will be informed and invited to participate in the study and asked for written informed consent. At study entry baseline demographics, obstetric and medical history will be recorded. Before cervical length measurement, women will be tested for fibronectin. Therefore a sample of cervicovaginal secretion will be taken by rotating a swab in the posterior vaginal fornix for 10 seconds. Analysis will be performed using the FullTerm TLi_IQ _system of Hologic [26].

Women with a cervical length below 10 mm and women with a cervical length between 10-30 mm in combination with a positive fibronectin test will be treated with tocolysis. These women are considered to be at high risk for preterm delivery. Women with a cervical length above 30 mm will be treated according to the discretion of the physician. Women with a cervical length between 10-30 mm and a negative fibronectin test are considered to be at low risk, they will enter a double blind trial in which they will be randomised by a central web-based system between tocolytics or placebo (1:1). Details of delivery and maternal assessments during pregnancy of all women are recorded in case record forms (CRF) that are accessible through a closed part of a central website. All data will be collected, coded and processed with adequate precautions to ensure patient confidentiality.

### Interventions

**Figure 1:**
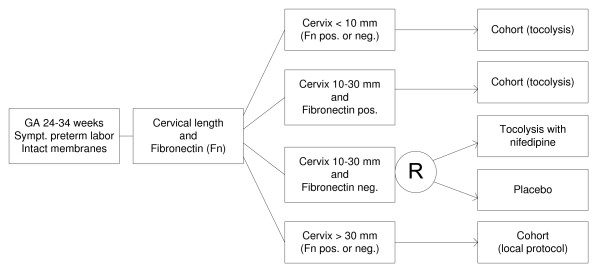
**Flow chart APOSTEL-I study, an overview of which patients in the APOSTEL-I cohort will be randomized based on their cervical length and fibronectin status**.

All high risk women will receive tocolytics according to local protocol. Fibronectin negative women with a cervical length between 10-30 mm will be randomly allocated to tocolytics or placebo (figure 1). Active treatment will be performed with the calcium channel blocking agent nifedipine with a total daily dose between 80 and 120 milligrams for 48 hours. Active drug and placebo will be administered orally at the same volume and rate. Corticosteroids will be administered at the discretion of the attending physician.

Progression of labor will be monitored every 12 hours by means of clinical observation and/or vaginal examinations for 48 hours. If labor seems inevitable in that period, study medication can be stopped and another tocolytic (excluding nifedipine) may be administered.

### Follow up of women and infants

Details of admission of newborns to the neonatal intensive care unit and neonatal complications will be recorded. Long term follow up is not part of this study.

### Outcome measures

The primary outcome measure is number of days to delivery truncated at 7 days after study entry. Secondary endpoints are neonatal mortality, neonatal morbidity, maternal morbidity (side effects of nifedipine), costs and health related quality of life.

### Statistical issues

#### Sample size

The randomised trial in our study will be a non-inferiority effectiveness trial. Based on our pilot study [25], we anticipate the probability on preterm birth within 7 days in the group of women with a negative fibronectine test and a cervical length 10-30 mm to be 5%. We need 220 fibronectin negative women in the cohort (110 per arm) to assure with 80% power that in case of non-inferiority the upper limit of the 95% one-sided CI for the difference in the proportion of preterm deliveries < 7 days will be within a prespecified boundary of 7.5%. According to our pilot we will need a cohort of approximately 660 women [25].

#### Data analysis

The results of the randomised trial will be analyzed according to the intention to treat principle. We will analyze costs and effects of a strategy based on fibronectin measurement and cervical length, in which only fibronectin positive women will be transferred to a perinatal center and/or and treated. Costs and effects of this strategy will be compared to a strategy in which these women will be transferred and treated merely based on a clinical diagnosis by e.g. vaginal examination and/or cervical length measurement.

#### Interim analysis

After 100 fibronectin negative, low risk women have entered the randomised trial, an interim analysis will be performed by an external independent safety committee. Interim analysis will be performed on the primary outcome only, e.g. percentage of preterm deliveries within 7 days after fibronectin testing. If there is a significant difference between the groups treated with tocolytics and placebo, the independent safety committee will decide about further unblinding of the interim results and (dis)continuation of the trial.

### Economic evaluation

The aim of the economic evaluation is to assess whether fibronectin testing as a triage for women with threatened preterm labor is cost-effective. For the cost analysis we distinguish cost within the antenatal period, during delivery and childbirth and within the postnatal period. As the study will be performed from a societal perspective, three cost categories will be included: direct medical costs, direct non-medical costs and indirect costs. Volumes of health care resource use during the index admission are measured prospectively alongside the clinical study in all participating centers as part of the CRF. Questionnaires addressing health related quality of life will be administered in a random subsample.

If the randomised trial indicates that a particular subgroup of women does not benefit from treatment with tocolytics, the economic analysis will be a cost-minimization analysis. If tocolysis and transferring low risk women would not be necessary, the costs of risk assessment (int.al. fibronectin testing) of the whole cohort of women will be compared to the potential cost saving. Furthermore, scenario analyses for relevant subgroups will be performed. Before analyzing the data from the study, a detailed cost analysis model will be developed.

The trial results will be incorporated in a diagnostic model to compare a risk assessment strategy for women with threatening preterm labor within current practice, which does not include additional risk assessment with fibronectin.

The study design will enable us to compare the costs and effects of the following strategies:

I. transfer and treat all women with threatening preterm labor

II. transfer and treat women based on the results of fibronectin measurement. As we also measure cervical length, we can also evaluate the following strategies:

III. transfer and treat women based on the results of cervical length measurement

IV. transfer and treat women based on a combination of cervical length measurement and fibronectin measurement

V. sequential combinations, for example first cervical length measurement, and fibronectin measurement in a part of that women, or vice versa.

In our cohort, we will obtain data on the moment of delivery in women treated with tocolytics and not treated with tocolytics. In addition to our cohort data, we will obtain data from medical literature on the occurrence of neonatal mortality and morbidity in relation to duration of pregnancy, both with and without treatment of corticosteroids. We will use this to calculate the expected neonatal mortality and morbidity for each of the five strategies mentioned above.

### Ethical consideration

This study has been approved by the ethics committee of the Academic Medical Centre Amsterdam (ref.no MEC 08/363).

## Discussion

In women with clinical signs of threatened preterm labor, fibronectin can discriminate between those at low risk and high risk for immediate preterm delivery. Since treatment of low risk women might be unnecessary, testing for fibronectin is thought to be a cost effective strategy. It may protect mother and fetus from possible side effects of tocolytics and corticosteroids, lessen burden of perinatal centers and decrease stress and anxiety for the families. So far, no randomised trial has shown beneficial effects of fibronectin testing for women presenting with threatened preterm labor, despite its high negative predictive value. Although the use of fibronectin tests for these women is already implemented in some clinics and even incorporated into guidelines [27], neither its safety as a triage instrument, nor its cost effectiveness has been established.

We decided to use a fixed protocol to translate the test results into a management strategy with nifedipine, to out rule the influence of individual physicians. Nifedipine has not yet been tested against placebo as a tocolytic drug, though both the Cochrane Collaboration [28] and the Dutch Society of Obstetrics and Gynaecology [2] recommend calcium antagonists as first choice. It is unlikely that using another tocolytic drug would lead to different outcomes of this trial, since fibronectin negative women have a low a priori probability of delivering within 7 days after the test. So even though we randomize on therapy level, this trial will evaluate fibronectin testing and cervical length measurement for women with threatened preterm labor.

## Competing interests

The authors declare that they have no competing interests.

## Authors' contributions

BWJM, JAMvdP, MAO, BO and JYV were involved in conception and design of the study. JYV, MAO and BWJM drafted the first manuscript. All authors mentioned in the manuscript are members of the APOSTEL I study group. They participated in the design of the study during several meetings and are local investigators in the participating centers. All authors edited the manuscript and read and approved the final draft.

## Pre-publication history

The pre-publication history for this paper can be accessed here:


